# Brain activation in parietal area during manipulation with a surgical robot simulator

**DOI:** 10.1007/s11548-015-1178-1

**Published:** 2015-04-07

**Authors:** Satoshi Miura, Yo Kobayashi, Kazuya Kawamura, Yasutaka Nakashima, Masakatsu G. Fujie

**Affiliations:** 1 Department of Modern Mechanical Engineering, Waseda University, Room 309, Bld. 59, 3-4-1 Okubo, Shinjuku, Tokyo, 169-8555 Japan; 2 Faculty of Engineering/Center for Frontier Medical Engineering/Graduate School, National University Corporation Chiba University, Science and Technology Building 1-511, 1-33, Yayoi, Inage, Chiba, 263-8522 Japan; 3 Yamamoto Laboratory, Kyushu University, West Building Zone 4, Office 434, 744, Motooka, Nishi-ku, Fukuoka, 819-0395 Japan

**Keywords:** Surgical robot, Surgical simulator, Tele-surgery, Brain activity measurement

## Abstract

***Purpose*:**

we present an evaluation method to qualify the embodiment caused by the physical difference between master–slave surgical robots by measuring the activation of the intraparietal sulcus in the user’s brain activity during surgical robot manipulation. We show the change of embodiment based on the change of the optical axis-to-target view angle in the surgical simulator to change the manipulator’s appearance in the monitor in terms of hand–eye coordination. The objective is to explore the change of brain activation according to the change of the optical axis-to-target view angle.

***Methods*:**

In the experiments, we used a functional near-infrared spectroscopic topography (f-NIRS) brain imaging device to measure the brain activity of the seven subjects while they moved the hand controller to insert a curved needle into a target using the manipulator in a surgical simulator. The experiment was carried out several times with a variety of optical axis-to-target view angles.

***Results*:**

Some participants showed a significant peak (*P* value = 0.037, *F*-number = 2.841) when the optical axis-to-target view angle was $75^{\circ }$.

***Conclusions*:**

The positional relationship between the manipulators and endoscope at $75^{\circ }$ would be the closest to the human physical relationship between the hands and eyes.

## Introduction

Robotic surgery offers the advantage of minimally invasive surgery, which can reduce both scarring and patient recovery time because the surgical manipulator is small and precise [1, 2]. Surgical robots are therefore used worldwide [3]. For example, 2000 da Vinci surgical robots have been sold worldwide, and these surgical robots were used in more than 278,000 cases prior to 2010 [4].

The method of operation when using a surgical robot mainly involves a master–slave arrangement, where the surgeon inserts the slave manipulators and an endoscope into the patient’s body, and then operates the slave manipulators using the master console. The surgeon controls the master console to move the slave manipulators within the patient’s body while simultaneously observing the operative field through the endoscope. In robotic surgery, the surgeon’s control depends on a combination of visual observation of the slaves via the endoscope and the proprioceptive senses of the operator’s hand via the stimulation from the nerves [5]. When the surgeon moves the master, the surgeon depends on proprioceptive feedback from his or her hand. Simultaneously, when the surgeon examines the slave’s movement, he or she depends on visual feedback about the slave’s movement from the endoscope. When the surgeon feels that the use of visual and proprioceptive senses to control the slaves and endoscope are as intuitive as his or her own hands and eyes, the instruments can be operated as intuitively as the surgeon can operate his or her own body.

Surgical robots must be designed to make the best use of the surgeon’s skill and experience when operating, and maximize the intuitiveness of operation. Although intuitiveness has been studied by many scientists in a variety of fields, the master–slave system used in surgical robots has some problems. One is how exactly the posture and position of the manipulator’s tips are synchronized between master and slave. In endoscopic surgery, the direction that the surgeon’s hand moves is contrary to the direction that the forceps moves through the endoscope in the monitor. The surgeon, in endoscopic surgery, does not feel that their visual and proprioceptive senses are agreement. However, robotic surgery resolves the problem of the agreement of the tip’s kinematics between surgeon and manipulator. Another issue is how the surgeon feels that the manipulator belongs to their body, because they are operating using the manipulator and endoscope instead of their hands and eyes. This feeling is called hand–eye coordination. When the trocar port point changes to a different part, the surgeon’s cognitive sense of hand–eye coordination changes. From the viewpoint of robotics, hand–eye coordination caused by the physical difference between the human body and the robot mechanism is known as embodiment [6–8]. The embodiment means the cognition which is strongly influenced by aspects of human body beyond the brain itself. Hand–eye coordination is one of the embodiment [7, 8].

Although the surgical robot must be designed with embodiment as a consideration, there is currently no good method for evaluating embodiment. Conventionally, engineers design surgical robots taking mechanical performance aspects into account, such as the time taken to complete a given task, and the average speed and curvature of a movement under test conditions [9]. These working scores are so useful, in fact, that the mechanical performance of surgical robots has improved considerably in recent years, but the improvement in the mechanical performance of a robot does not necessarily represent the embodiment that the user feels.

In the field of cognitive neuroscience, many related studies have reported that the intraparietal sulcus is the specific brain area that is important in the function of embodiment. Some reported that the intraparietal sulcus shows how strongly a human perceives that a tool belongs to their body [10, 11]. In Iriki’s experiment, the intraparietal sulcus of the macaque changes before and after tool-use. Iriki reported that this result occurred because the macaque perceives the tool as belonging to its body [12, 13]. The intraparietal sulcus was also activated as the macaque saw its hand in the virtual space [14] and was measured in real time while using the tool with positron emission computerized tomography (PET) [15]. The function of the intraparietal sulcus is reported to be applicable to not only macaques but also humans [16, 17]. In addition, activity in the intraparietal sulcus has been found using not only f-MRI but also f-NIRS [18–20]. This related research has often been reported in the cognitive science field, but very few attempts have been made to apply these findings to robotics design in the field of engineering.

**Fig. 1:**
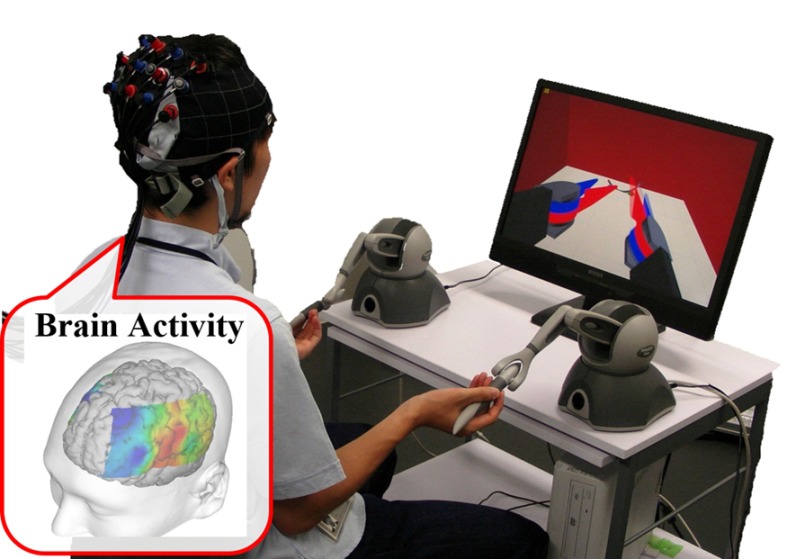
Proposed method. We measured the user’s brain activity using a f-NIRS brain activity measurement device during a simulation involving virtual robot surgery. The user moved the hand controller to manipulate the virtual arm

**Fig. 2:**
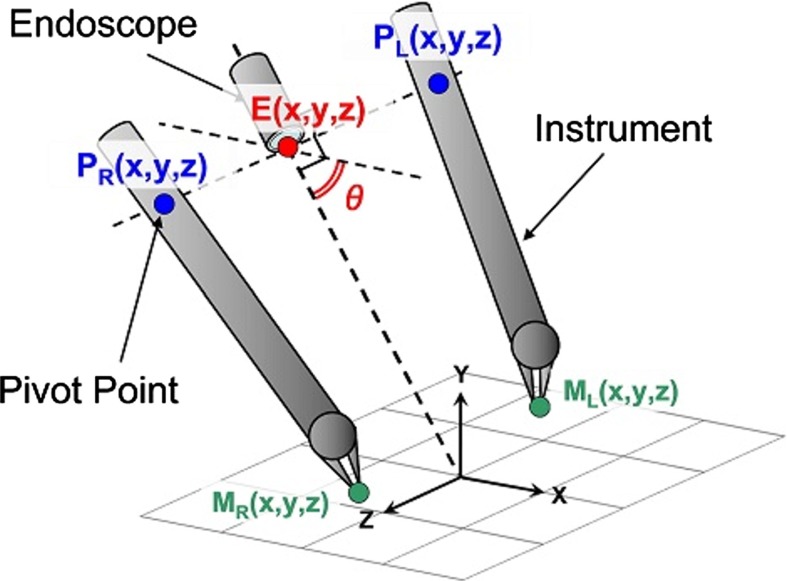
Hand–eye coordination configuration, consisting of the two instruments and the endoscope. The two *pivot points* are in parallel with the tip of the endoscope. The experiment is carried out for various values of the optical axis-to-target view angle $({\uptheta })$

In this paper, we validate the feasibility of a method to qualify the embodiment caused by the physical difference between master–slave surgical robots by measuring the activation of the intraparietal sulcus in the user’s brain activity during surgical robot manipulation as shown in Fig. 1. We measured the brain activity while the user controlled the manipulator on a surgical simulator, and determined an optimal robot design. In the experiment, to change the manipulator’s appearance in the monitor in terms of hand–eye coordination, we changed the endoscope viewpoint such as optical axis-to-target view, as shown in Fig. 2. We show the change of embodiment as change of the optical axis-to-target view angle, and explore the change in brain activation as a function of the change of the optical axis-to-target view angle.

## Method

### Experimental setup

**Fig. 3:**
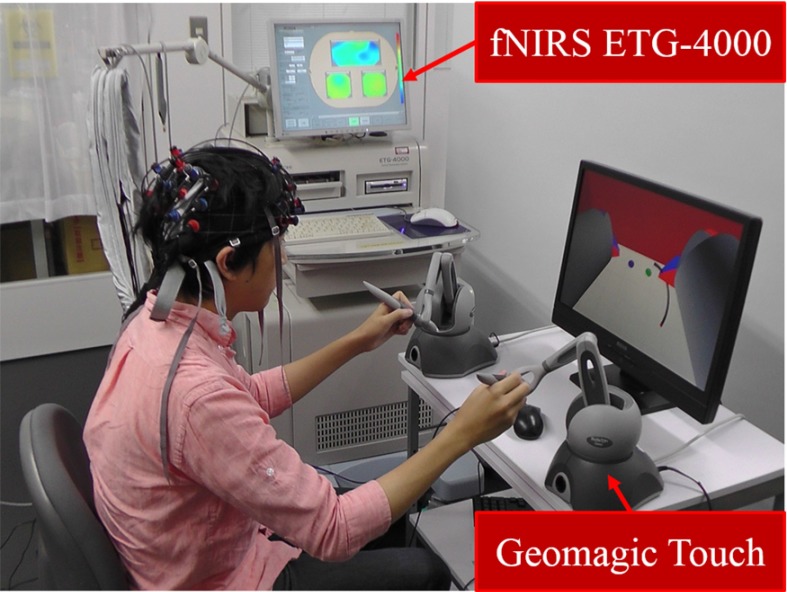
Experimental setup. The participant moved the two Geomagic Touch hand controllers to control the two virtual manipulators. We measured the participant’s brain activity using the f-NIRS brain function measurement device

We performed brain imaging using a functional near-infrared spectroscopic topography (f-NIRS) device shown in Fig. 3. F-NIRS is a relatively new brain imaging technique in which brain activity is indicated by the relative change in the concentrations of oxygenated hemoglobin concentration and deoxygenated hemoglobin concentration. F-NIRS transmits the near-infrared light to the subject’s head and receives the backed near-infrared light via the cerebral cortex. In the cerebral cortex, the near-infrared light is absorbed in the oxygenated hemoglobin concentration of the vessels in the brain which changes in proportion as the brain activation. F-NIRS shows the oxygenated hemoglobin concentration as the brain activation through the change of the near-infrared light between before and after passing the subject’s head. We used f-NIRS (ETG-4000; Hitachi Medico Co., Tokyo, Japan) to evaluate the activity around the intraparietal sulcus. Thus, the higher the changes in the oxygenated hemoglobin concentration around the intraparietal sulcus become, the more agreement of physical difference between master–slave the participant will feels because the intraparietal sulcus has the function of the embodiment [10, 11]. Whereas f-NIRS does have inferior resolution performance when compared to f-MRI, it allows brain activity measurements to be performed without the use of magnetic fields [21, 22]. f-NIRS takes advantage of the measurement during controlling the robots and machines, including master–slave system such as the automobile and artificial limbs [23, 24]. Additionally, when using f-MRI the participant needs to lie down, but when using f-NIRS the participant can perform body movement tasks [21, 22]. Finally, f-NIRS is reasonably compact.

**Fig. 4:**
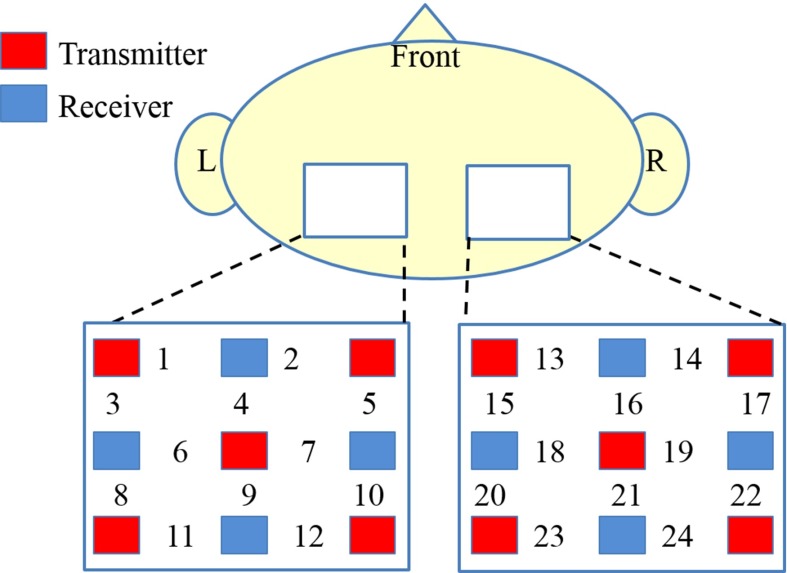
Cap position, the arrangement of the 18 photodiodes and the locations of the 24 measurement channels. The *red and blue squares* indicate the NIRS light transmitters and receivers, respectively. The *numbers* indicate the channels

We used two 3 $\times$ 3 matrixes of photodiodes, consisting of 10 light transmitters and 8 receivers for the measurements, as shown in Fig. 4. The blood oxygen level was measured in the 30-mm area between each transmitter and receiver pair. In the parietal area, the two-scalp setup consisted of nine photodiodes forming 12 measurement channels. The intraparietal sulcus is positioned at the intraparietal area. To measure the activity of the intraparietal sulcus, we identified the channel on the participant’s head that corresponded to that brain area using the following procedure.

First, we measured the three-dimensional (3D) coordinates of each point on the participant’s head using a 3D position measurement device (3D digitizer; PATRIOT Digitizer; Polhemus; USA). Next, we compared the 3D coordinates of each of these points on the participant’s head with a standard brain model using platform for optical topography analysis tools (POTATO) combined with MATLAB to identify the channel that was above the intraparietal sulcus [25].

While we measured the brain activity of each participant, they moved a hand controller (Geomagic Touch, Geomagic, Raleigh, NC, USA) to control the virtual arm in the surgical simulator (Fig. 3). The simulation was presented to the user on a 24-inch liquid crystal display (LCD) monitor with a vertical refresh rate of 60 Hz. The time course of the stimulus presentation was controlled using a personal computer (PC). The participants set the monitor position to be perpendicular to their line of sight at their own discretion.

**Fig. 5:**
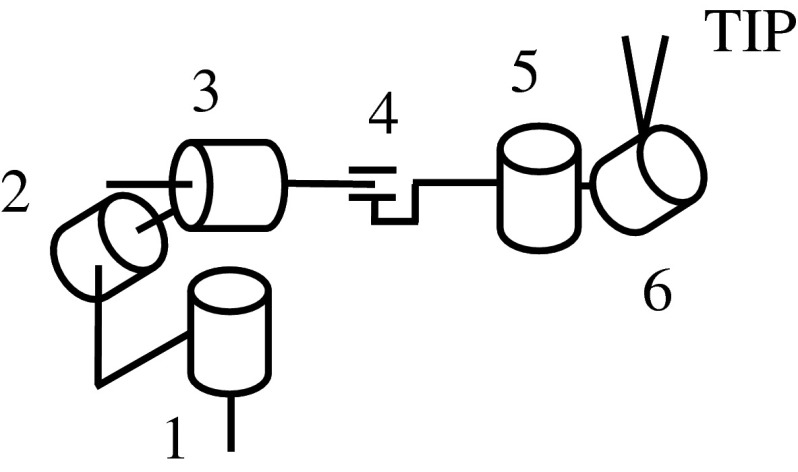
Denavit–Hartenberg skeleton [26]. The manipulator has six DOF

**Fig. 6:**
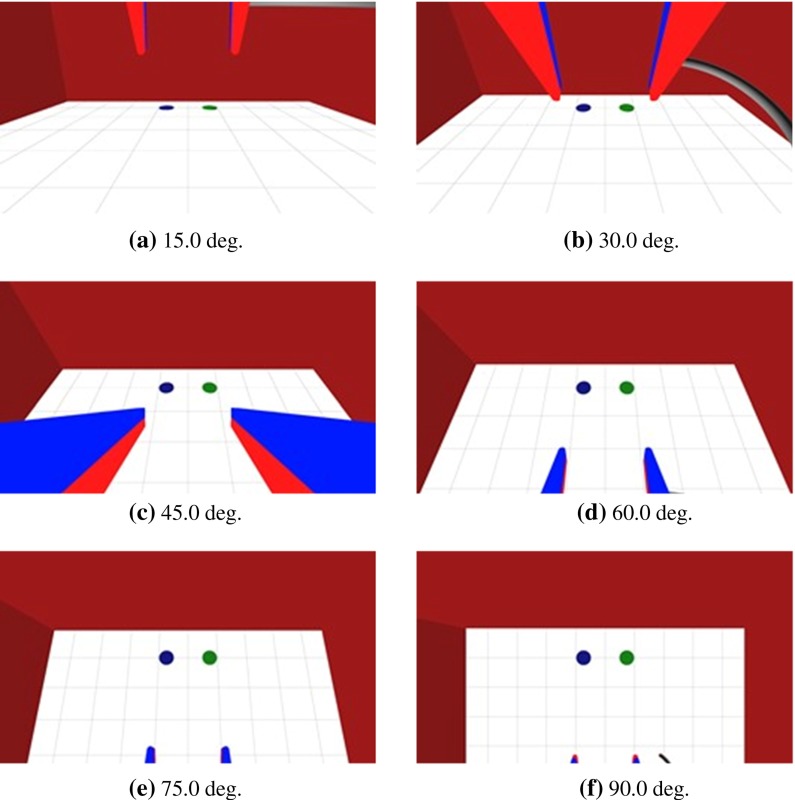
Experimental condition. The optical axis-to-target view angle of the endoscope changes by $15^{\circ }$

The virtual manipulator has six degrees-of-freedom (DOF), as shown in Fig. 5. Figure 6 shows the simulator with blue and green circle targets on the white plane around the red wall, and a gray virtual curved needle with two virtual manipulators. The virtual arm mechanism is similar to that of the da Vinci. The position and posture of the tip was synchronized between the virtual arm and the hand controller while the user operated the foot switch. The manipulator can grip the curved needle when the participant pushes the stylus button.

### Experimental conditions

The experimental conditions were based on the optical axis-to-target view angle because we examined a hand–eye coordination configuration consisting of two hands and eyes. The optical axis-to-target view angle was determined such that the endoscope downward posture against the plane as shown in Fig. 2. We conducted tests using a total of six different optical axis-to-target view angles, which were $15^{\circ }, 30^{\circ }, 45^{\circ }, 60^{\circ }, 75^{\circ }$ and $90^{\circ }$, as shown in Fig. 6. At each optical axis-to-target view angle, the two pivot points were in parallel with the tip of the endoscope. The distance between the endoscope and the origin stayed constant.

The task was manipulation of the virtual arm to insert the curved needle. The participant moved the hand controller to control the virtual arm while operating the foot switch. The participant inserted the needle from the left green circle point into the right blue circle point, as shown in Fig. 6. When the participant finished the manipulation task, they then released the needle from the manipulator. We confirmed the release of the needle and stopped the measurement. Under the initial experimental conditions, the simulator started the condition such that the manipulator gripped the needle and positioned it in parallel with the plane. At the start of the task, the participant positioned the stylus in parallel with the plane while operating the foot switch and synchronizing the virtual manipulator. The task was performed this way because the posture of the virtual manipulator corresponded to the posture of the stylus against the plane.

Five healthy adults (four men and one woman; mean age of 24 years; age range of 23–26 years; six right-handed and one left-handed) participated in the experiment. All participants had normal or corrected-to-normal vision. The participants were informed about the measurement of their brain activity and the purpose of the experiment. Informed consent was obtained from all individual participants included in the study. The experiments were conducted in accordance with the Declaration of Helsinki and were approved by the Waseda University Institutional Review Board (No. 2013-201). All participants were students at Waseda University, Japan, and were not surgeons. We considered them to be appropriate participants for determination of human cognitive function based on measurements of their brain activity.

### Experimental procedure

First, we placed the cap of the imaging device on the participant’s head. Next, the participant trained by performing a few trials under each experimental condition before starting the experiment. Third, the participant performed five trials for insertion of the curved needle into the target under each of six conditions. At the start of the measurement sequence, the imaging device scanned the oxygenated hemoglobin concentration in the participant’s brain for 10.0 s. When the scan was complete, the display showed the surgical simulator automatically, and the participant started the task. During each measurement session, the participant tried to maintain the same posture and minimize body movement. The order of the experimental conditions was random.

We corrected the raw data using the following procedures. First, the raw data were digitally low-pass filtered at 0.10 Hz to remove the measurement noise. Next, a baseline correction was performed to remove the linear trend in the hemoglobin concentration. We fitted a linear function to the data points sampled in the 5-s intervals before and after the onset of each task period. Because the raw data for f-NIRS consist of relative values, we could not compare the data from the participants or that of the channels directly.

## Results

The mean and standard deviation of the oxygenated hemoglobin concentration data in all subjects is $0.00399\pm 0.06431$ mMmm. The maximum of all oxygenated data is 0.39403 mMmm, and the minimum is $-0.4214$ mMmm. All the channel shows no significant using ANOVA, one-way analysis of variance (*P* value = 0.68843, *F*-number = 0.83077) for null hypothesis.

**Fig. 7:**
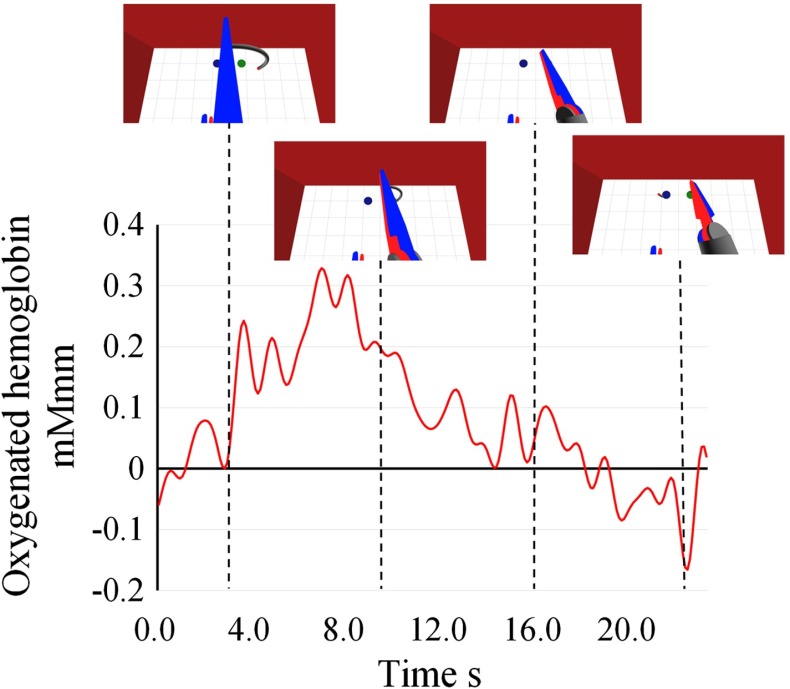
Longitudinal data of oxygenated hemoglobin concentration in the channel that corresponded with the intraparietal sulcus during manipulation task for one participant. The oxygenated hemoglobin concentration changed depending on the phase of the manipulation

Figure 7 shows the longitudinal data of the oxygenated hemoglobin concentration from the channel that corresponded with the intraparietal sulcus during the curved needle insertion task for one participant. The oxygenated hemoglobin concentration changed depending on the phase of the needle insertion process. The oxygenated hemoglobin concentration increased when the needle was close to the green circle and peaked when the tip of the needle touched the circle. The oxygenated hemoglobin concentration decreased during insertion of the curved needle until the needle tip appeared.

**Fig. 8:**
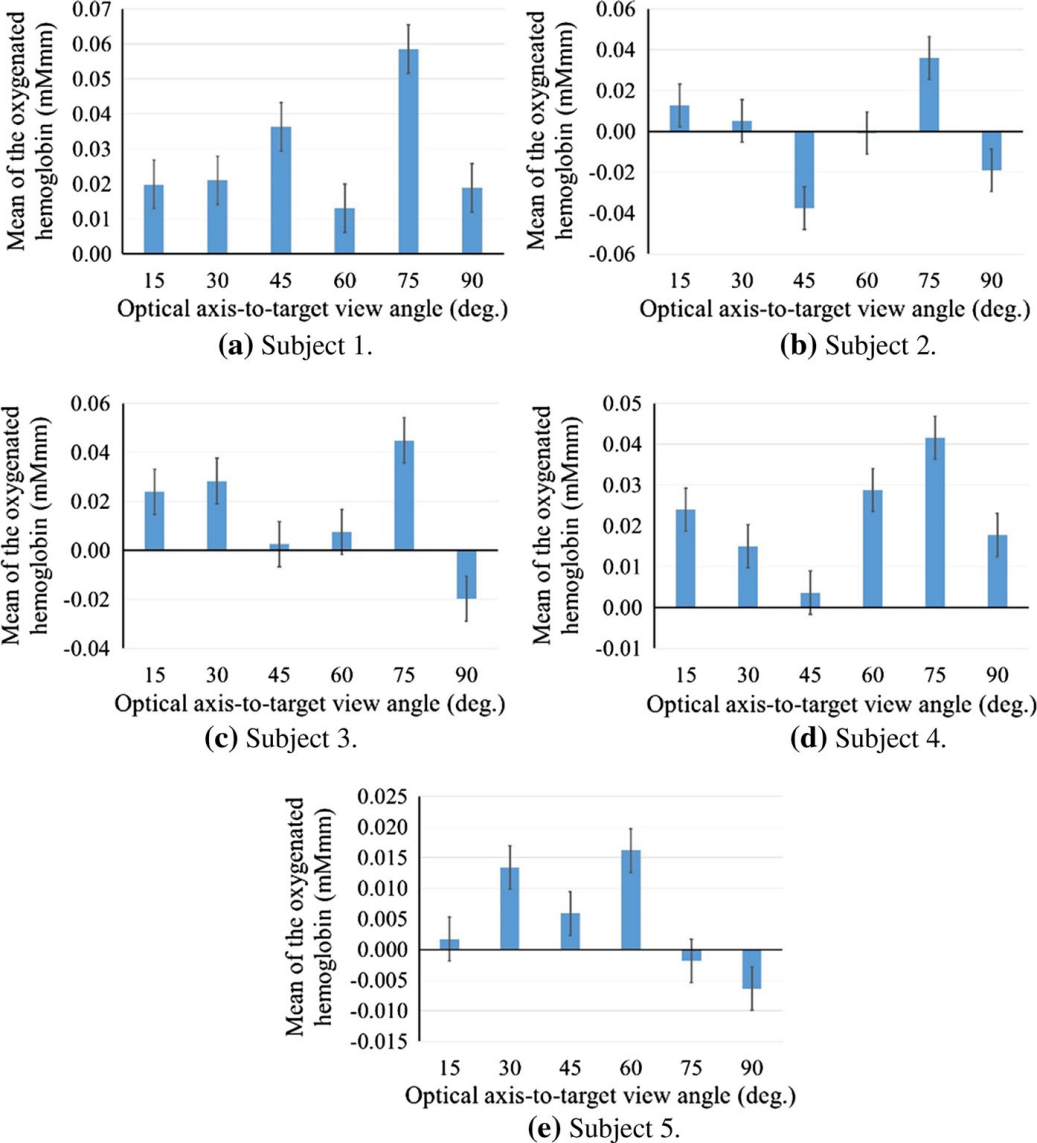
Mean value of the oxygenated hemoglobin concentration versus the optical axis-to-target view angle for each participant. *Error bar* shows the SD

**Fig. 9:**
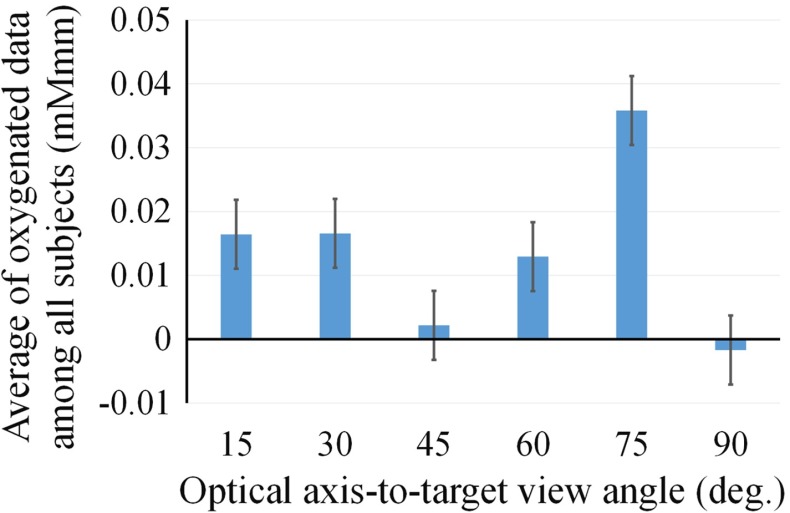
Average of the optical axis-to-target view angle at the most brain activated among all trials in all participants. *Error bar* shows SD

Figure 8 shows the mean value of the oxygenated hemoglobin concentration against the optical axis-to-target view angle for each participant. The mean was calculated during the task period over five trials. We used ANOVA for statistical testing and found that the data showed significant differences (*P* value = 0.037, *F*-number = 2.841). Figure 9 shows the average of the optical axis-to-target view angle at the most brain activated among all trials in all participants.

## Discussion

The mean of the oxygenated data is only 0.00399, but standard deviation is 0.06431 mMmm because oxygenated data fluctuated widely according to the phase of the manipulation. The Fig. 7 shows that the brain activation changed widely during the manipulation. The trend observed in Fig. 7 suggests that the intraparietal sulcus can be used to determine the user’s introspection for physical human–robot correspondence. This is because the intraparietal sulcus activated depending on whether the tip of the virtual arm appeared. For the user to perceive the tool as part of their body, it would be necessary to synchronize the posture and position of the tip between master and slave. In Fig. 7, during the time where the curved needle was showing, the oxygenated hemoglobin concentration increased. In contrast, when the curved needle was hidden, the oxygenated hemoglobin concentration decreased. During the period where the participant could look at the tip, they perceived the manipulator as being part of their body.

**Fig. 10:**
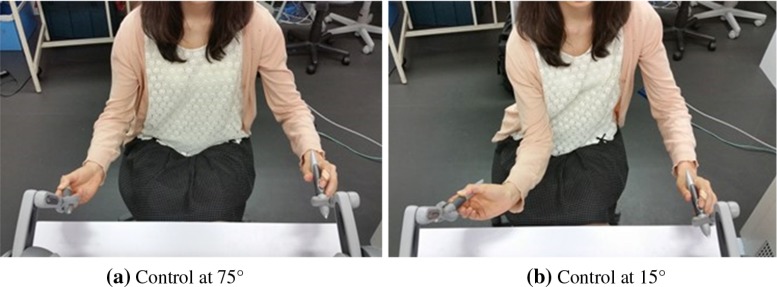
Differences in rotation of the participant’s wrist. In **a**, with the control at $75^{\circ }$, the participants manipulated the virtual arm to insert the curved needle by rotating their wrist only. However, in **b**, with the control at $15^{\circ }$, the participants manipulated the arm by bending their elbow

Figure 8 shows that the brain activates the most significantly at $75^{\circ }$ in four subjects of five. In Fig. 9, the average of the oxygenated hemoglobin concentration is the most significantly at $75^{\circ }$. The positional relationship between the manipulators and endoscope at $75^{\circ }$ would be the closest to the human physical relationship between hands and eyes because the positional relationship between the subject’s eyes and the screen is at $75^{\circ }$. The view angle of the endoscope in the simulator matches the relationship between the eyes and the screen so that the operator would have better hand–eye coordination. At angles of $1^{\circ }$ and $30^{\circ }$, the brain activated more than at angles of $45^{\circ }$ and $60^{\circ }$. The participants can perceive the depth because at this angle, the depth is easily visible. However, the participants rotated their wrists with difficulty. Figure 10 shows the difference between rotating the wrist at $15^{\circ }$ and at $75^{\circ }$. At $75^{\circ }$, each participant manipulated the virtual arm to insert the curved needle by rotating their wrists only. However, at $15^{\circ }$, the participants operated the virtual arm to insert the needle by bending their elbows. The ease of rotation of the wrist would have had an effect on the difference in brain activity observed between $15^{\circ }$ and $75^{\circ }$. The brain activity at $75^{\circ }$ is higher than that at $15^{\circ }$ because the participants could rotate their wrists more easily at $75^{\circ }$ than at $15^{\circ }$. The case that the kinematics of the manipulator is adjusted to move the subject’s arm easily at $15^{\circ }$, the oxygenated hemoglobin concentration would increase. At $45^{\circ }$, $60^{\circ }$ and $90^{\circ }$, the brain activation was low. At $90^{\circ }$, the participants can rotate their wrists easily, but it is the most difficult angle at which to perceive the depth. The view at $45^{\circ }$ marks a halfway point for ease of rotation of the wrist and perception of the depth. Therefore for intuitive control, it is important to support both ease of rotation of the wrist and perception of the depth. In future work, we will measure the user’s brain activity and the movement of the arm to compare the cognitive function with a muscle–skeletal model.

In this paper, the optical axis-to-target was changed within only a range. Under the several range changed, the brain activation should be measured. The other parameters such as the distance between stereo scopes and lighting direction would also have an effect on the brain activation. In addition, the DOF between the master and slave was different. In future, the brain activation should be validated in the consideration of these conditions. Furthermore, not only the indirect manipulation system such as the master–slave, but it is necessary to see the f-NRIS measurement when the user is directly manipulating objects naturally to establish a comparative baseline.

## Conclusion

In this study, we validated an evaluation method to qualify the embodiment caused by the physical difference between master–slave surgical robots by measuring the activation of the intraparietal sulcus in the user’s brain activity during surgical robot manipulation. The objective was to explore the change of the brain activation according to the change of the optical axis-to-target view angle. We measured the brain activity of the participants while they moved a hand controller to control a virtual arm using a variety of optical axis-to-target view angles. We found that some participants showed a significant peak (*P* value = 0.037, *F* number = 2.841) in brain activity at $75^{\circ }$. We concluded that the positional relationship between the manipulators and endoscope at $75^{\circ }$ would be the closest to the human physical relationship between the hands and eyes. The proposed method and the results obtained in this paper are likely to have a spillover effect into artificial arms and other master–slave systems, such as infrastructure building robots.
